# Age-stratified toxicity patterns of CDK4/6 inhibitors in older women with breast cancer: Disproportionality analysis from the FAERS database

**DOI:** 10.1016/j.breast.2025.104687

**Published:** 2025-12-29

**Authors:** Bahadır Köylü, Buğra Han Esen, Cevat İlteriş Kıkılı, Fatih Kemik, Nazan Demir, Şeyda Gündüz, Şahin Laçin, Deniz Tural, Didem Tunalı, Gülistan Bahat, Fatih Selçukbiricik

**Affiliations:** a Koç University, School of Medicine, Department of Internal Medicine, Division of Medical Oncology, Istanbul, Turkiye; b Koç University, School of Medicine, Istanbul, Turkiye; c Istanbul University, Istanbul Faculty of Medicine, Department of Internal Medicine, Division of Geriatrics, Istanbul, Turkiye

**Keywords:** Cyclin-dependent kinase inhibitors, Breast neoplasms, Pharmacovigilance

## Abstract

**Background:**

Despite their benefits, the safety of cyclin-dependent kinases 4 and 6 (CDK4/6) inhibitors in older patients remains underexplored. We aimed to investigate the toxicities associated with CDK4/6 inhibitors in subgroups of older patients, through analysis of the FAERS database.

**Methods:**

In this retrospective pharmacovigilance analysis, we identified 49,223 females with breast cancer (aged 18–100 years), in which a CDK4/6 inhibitor (palbociclib, ribociclib, or abemaciclib) was recorded as the primary suspect drug. Patients were stratified into four age groups: <65, 65–74, 75–84, and ≥85 years. Disproportionality analysis using the reporting odds ratio method was conducted to detect positive disproportionality signals between individual adverse events and CDK4/6 inhibitors. We performed age-stratified multivariate analyses to detect age-related differences.

**Results:**

Acute renal failure and interstitial lung disease associated with abemaciclib were reported more frequently in geriatric age subgroups, while gastrointestinal and hematologic adverse events showed a declining reporting frequency with advancing age. Significant age-related increases in the odds of reporting dementia, hearing and vestibular disorders, lens disorders, arthritis, thrombotic events, and central nervous system hemorrhagic complications were identified in palbociclib-treated patients. Ribociclib showed increased reporting of acute renal failure, chronic kidney disease, cardiac arrhythmias, and ischemic heart disease in geriatric age subgroups, whereas the reporting frequency of liver-related adverse events declined with advancing age.

**Conclusions:**

Older adults receiving CDK4/6 inhibitors experience higher rates of renal, pulmonary, cardiac, and neurocognitive toxicities, with abemaciclib linked to renal and pulmonary, palbociclib to neurological and thrombotic/hemorrhagic, and ribociclib to renal and cardiac adverse events.

## Highlights

• Abemaciclib is more frequently associated with acute renal failure and ILD in 65+.
• Palbociclib shows a strong association with dementia, requiring attention.
• 65+ patients experience more renal and cardiac adverse events with ribociclib.

## 1 Introduction

Breast cancer is the leading cause of cancer globally among females, accounting for 24 % of all cancer cases and 15 % of cancer-related deaths [1]. Its incidence, particularly of hormone receptor-positive (HR+) disease, has been steadily increasing, primarily driven by increasing obesity prevalence and declining fertility rates [2,3]. As the global population ages, geriatric patients now represent a substantial proportion of newly diagnosed breast cancer cases, with approximately one-third aged ≥70 years and one-ninth aged ≥80 years [4]. Future projections indicate a more pronounced global burden of breast cancer in older female populations [5].

Many cyclins and cyclin-dependent kinases (CDKs) play fundamental roles in cell-cycle regulation by forming heterodimer complexes that phosphorylate target genes [6]. The activation of CDKs by cyclins is counterbalanced by CDK inhibitors such as p16^INK4a^, p15^INK4b^, and p18^INK4c^, which function as tumor suppressors by preventing inappropriate cell proliferation through timely inhibition [7]. Genetic alterations in these regulatory proteins drive uncontrolled cell proliferation in breast cancer [8]. The selective CDK4/6 inhibitors (CDK4/6is)–palbociclib, ribociclib, and abemaciclib– are potent agents that arrest cell-cycle progression and demonstrate clinical efficacy in HR+ breast cancer, particularly when combined with anti-estrogen therapy [8].

Despite their clinical benefits, CDK4/6is are associated with various side effects, including cytopenias, diarrhea, elevated liver enzymes, and cardiotoxicity, which can compromise quality of life and contribute to increased frailty in older adults [9,10]. However, the safety of CDK4/6is in older adults remains underexplored, as this population is mostly underrepresented in randomized clinical trials (RCTs) [11]. Our objective was to investigate the toxicity profiles of CDK4/6is in older patients, through analysis of the U.S. Food and Drug Administration Adverse Event Reporting System (FAERS) database.

## 2 Methods

### 2.1 Data source and study design

This retrospective pharmacovigilance study utilized data from the FAERS, a spontaneous reporting database that supports post-marketing safety surveillance for approved drugs and biologics. FAERS collects adverse event reports from healthcare professionals, consumers, and pharmaceutical manufacturers, capturing key clinical and administrative information through structured datasets. These include patient demographics (DEMO), reported drugs (DRUG), adverse events (REAC), therapy timelines (THER), outcomes (OUTC), report source (RPSR), and treatment indications (INDI). Further information on FAERS is available via the FDA website (https://fis.fda.gov/extensions/FPD-FAQ/FPD-FAQ.html).

### 2.2 Case selection and exposure definition

We extracted all adverse event reports submitted between January 1, 2015 and March 31, 2025. Deduplication was performed in accordance with FDA guidance: for identical CASEIDs, the most recent report (based on FDA_DT) was retained; if both CASEID and FDA_DT were the same, the report with the higher PRIMARYID was kept. From the resulting 14,208,447 unique cases, we identified female patients with breast cancer in which a CDK4/6i, palbociclib, ribociclib, or abemaciclib, was recorded as the “primary suspect drug” in the ROLE_COD field. We excluded cases where the reported age was <18 or >100 years, or where age was missing. The final analytic sample comprised 49,223 cases.

### 2.3 Adverse event classification

We evaluated the safety profile of CDK4/6is using all Standardised MedDRA Queries (SMQs) available in MedDRA version 28.0. SMQs are pre-defined groupings of MedDRA Preferred Terms (PTs) designed to facilitate standardized identification of adverse event clusters with shared clinical relevance. Each SMQ consists of a set of PTs curated to represent a specific medical condition or syndrome, and many SMQs are further categorized into broader groupings based on pathophysiological or organ-specific domains. This hierarchical structure allows certain SMQs to serve as subsets of broader, composite SMQs. In our analysis, all available SMQs were mapped to reported adverse events in the FAERS REAC dataset using PT codes. A full listing of included SMQs, along with their respective PT groupings and hierarchical classifications, can be seen at: https://tools.meddra.org/wbb/

### 2.4 Age stratification and covariate definition

Patients were stratified into four age groups: <65, 65–74, 75–84, and ≥85 years. In addition to age and CDK4/6i subtype, we identified three key covariates for adjustment: the presence of concomitant endocrine therapy, chemotherapy, and other targeted therapies. These were identified through the DRUG dataset by evaluating agents reported with ROLE_COD values indicating co-administration (i.e., “secondary suspect”, “concomitant”, or “interacting”). The International Nonproprietary Names of these drugs included in this analysis were sourced from the WHO Collaborating Centre for Drug Statistics Methodology under the classification “L01 ANTINEOPLASTIC AGENTS”. Comprehensive lists of the medications analyzed are available in Tables S1, S2, and S3.

### 2.5 Statistical analysis

Key demographic and clinical characteristics, including age, clinical outcomes, reporter type, treatment year, CDK4/6i used, and additional therapies, were first summarized.

All SMQs defined in MedDRA version 28.0 were included in disproportionality analysis using the Reporting Odds Ratio (ROR) method, as described in Table S4. This analysis was conducted to detect positive disproportionality signals (defined as a lower 95 % confidence interval [CI] of the ROR exceeding 1) between individual SMQs and CDK4/6is. The complete list of SMQs, along with their corresponding disproportionality analysis results is presented in Fig. S1. SMQs with positive disproportionality signals were further assessed through univariate logistic regression, conducted separately for each CDK4/6i (abemaciclib, palbociclib, and ribociclib). Covariates included age category (with <65-year group as the reference group), endocrine therapy, chemotherapy, and co-exposure to other targeted therapies. Variables with a P value < 0.05 in univariate models, along with age category (which was included in all models by design), were entered into multivariate logistic regression models to estimate adjusted odds ratios (aORs) and corresponding 95 % CIs. To ensure model reliability and avoid overfitting, only SMQs with at least 60 reported events were retained in the final multivariate analyses, based on the commonly accepted rule of requiring ≥10 outcome events per included variable (six covariates in total) [12]. This threshold was applied as a pragmatic safeguard against sparse-data instability, which can lead to inflated or highly variable effect estimates and overly optimistic or poorly calibrated confidence intervals when the number of outcome events is small relative to the number of variables estimated.

All analyses were conducted using R version 4.3.1, with two-sided P-values <0.05 considered statistically significant.

As the FAERS database is publicly available and anonymized, no ethics committee approval or informed consent was required for this study.

## 3 Results

A total of 49,223 female breast cancer cases with reported adverse events associated with CDK4/6is were identified, including 4918 cases (10.0 %) for abemaciclib, 38,002 (77.2 %) for palbociclib, and 6303 (12.8 %) for ribociclib (Table 1). The median (IQR) age across all cases was 66 (57–74) years, with patients aged ≥65, ≥75, and ≥85 years constituting 54.2 % (n = 26,669), 23.2 % (n = 11,398), and 4.4 % (n = 2163) of the total cohort, respectively.

**Table 1:** Demographics and clinical characteristics in female patients with breast cancer reporting adverse events with CDK4/6 inhibitors in the FAERS database.

Clinical Characteristics	Abemaciclib (n = 4918)	Palbociclib (n = 38,002)	Ribociclib (n = 6303)	All (n = 49,223)
**Age, median (IQR)**	62 (53–70)	67 (58–75)	61 (50–70)	66 (57–74)
18–64 years, n (%)	2894 (58.9 %)	15,885 (41.8 %)	3775 (59.9 %)	22,554 (45.8 %)
65–74 years, n (%)	1263 (25.7 %)	12,438 (32.7 %)	1570 (24.9 %)	15,271 (31.0 %)
75–84 years, n (%)	621 (12.6 %)	7784 (20.5 %)	830 (13.2 %)	9235 (18.8 %)
+85 years, n (%)	140 (2.9 %)	1895 (5.0 %)	128 (2.0 %)	2163 (4.4 %)
**Additional therapies, previous/concomitant**				
Endocrine therapy, n (%)	1613 (32.8 %)	16,494 (43.4 %)	2965 (47.0 %)	21,072 (42.8 %)
Chemotherapy, n (%)	33 (0.7 %)	230 (0.6 %)	105 (1.7 %)	368 (0.8 %)
Other targeted therapies, n (%)	45 (0.9 %)	395 (1.0 %)	131 (2.1 %)	571 (1.2 %)
Immune checkpoint inhibitors, n (%)	5 (0.1 %)	13 (0.03 %)	4 (0.06 %)	22 (0.04 %)
**Time to onset (days), median (IQR)**	32 (10–115)	93 (21–341.5)	66 (16–253)	73 (19–280)
**Dechallenge**				
Applied, n (%)	1654 (33.6 %)	7379 (19.4 %)	2147 (34.1 %)	11,180 (22.7 %)
Positive, n (%)	1313 (26.7 %)	4755 (12.5 %)	1779 (28.2 %)	7847 (15.9 %)
Negative, n (%)	341 (6.9 %)	2624 (6.9 %)	368 (5.8 %)	3333 (6.8 %)
Not applied, n (%)	495 (10.1 %)	5257 (13.8 %)	1701 (27.0 %)	7453 (15.1 %)
Unknown, n (%)	2769 (56.3 %)	25,366 (66.8 %)	2455 (39.0 %)	30,590 (62.2 %)
**Outcomes**				
Serious outcome	2669 (54.3 %)	20,638 (54.3 %)	5823 (92.4 %)	29,130 (59.2 %)
Death	373 (7.6 %)	4943 (13.0 %)	1594 (25.3 %)	6910 (14.0 %)
Hospitalization	1274 (25.9 %)	6218 (16.4 %)	1998 (31.7 %)	9490 (19.3 %)
Life-threatening	110 (2.2 %)	321 (0.8 %)	289 (4.6 %)	720 (1.5 %)
Disability	55 (1.1 %)	170 (0.5 %)	108 (1.7 %)	333 (0.7 %)
Other serious outcome	1364 (27.7 %)	13,417 (35.3 %)	3600 (57.1 %)	18,381 (37.3 %)

IQR, interquartile range; NA, not applicable.

**Fig. 1:**
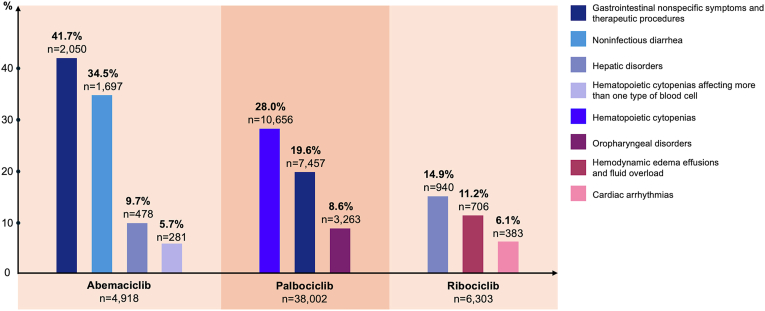
Overall reporting frequencies of the most commonly reported SMQs with positive disproportionality signals across different CDK4/6 inhibitors. (Only SMQs with >5 % reporting frequency are demonstrated in the figure.)

For abemaciclib, the most frequently reported SMQ was “gastrointestinal nonspecific symptoms and therapeutic procedures” (ROR = 3.99, 95 % CI 3.83–4.15), with a reporting frequency of 41.7 % across all age subgroups (Fig. 1). This was followed by “noninfectious diarrhea” (34.5 %) (ROR = 7.23, 95 % CI 6.85–7.63), “hepatic disorders” (9.7 %) (ROR = 1.75, 95 % CI 1.61–1.89), and “hematopoietic cytopenias affecting more than one type of blood cell” (5.7 %) (ROR = 1.45, 95 % CI 1.29–1.64). For palbociclib, the leading SMQ was “hematopoietic cytopenias” (28.0 %) (ROR = 1.88, 95 % CI 1.84–1.92), followed by “gastrointestinal nonspecific symptoms and therapeutic procedures” (19.6 %) (ROR = 1.16, 95 % CI 1.14–1.19) and “oropharyngeal disorders” (8.6 %) (ROR = 1.56, 95 % CI 1.50–1.62). For ribociclib, “hepatic disorders” was the most frequently reported SMQ (14.9 %) (ROR = 1.68, 95 % CI 1.30–1.50), followed by “hemodynamic edema effusions and fluid overload” (11.2 %) (ROR = 3.25, 95 % CI 2.95–3.58), and “cardiac arrhythmias” (6.1 %) (ROR = 1.81, 95 % CI 1.61–2.04).

In abemaciclib group, multivariate analysis revealed higher odds of reporting acute renal failure in patients aged 65–74 years (OR = 2.29 [95 % CI 1.63–3.21]; p < 0.0001) and 75–84 years (OR = 2.06 [95 % CI 1.33–3.13]; p = 0.0010) relative to those <65 years (Table 2). All geriatric age subgroups demonstrated increased odds of reporting interstitial lung disease (ILD), with the highest odds in patients aged 75–84 years (OR = 2.39 [95 % CI 1.64–3.42]; p < 0.0001). Eosinophilic pneumonia was elevated only in patients aged 75–84 years (OR = 2.04 [95 % CI 1.04–3.80]; p = 0.030), while an increase in thromboembolic events was observed only in patients aged 65–74 years (OR = 1.64 [95 % CI 1.21–2.23]; p = 0.001). Conversely, “gastrointestinal nonspecific symptoms and therapeutic procedures” was less frequently reported in all geriatric age subgroups, with the lowest odds in ≥85-year group (OR = 0.70 [95 % CI 0.49–0.99]; p = 0.047). Similarly, geriatric age subgroups had progressively lower odds of reporting “hematopoietic cytopenias affecting more than one type of blood cell” with advancing age, with the most pronounced reduction in ≥85-year group (OR = 0.09 [0.00–0.41]; p = 0.018).

**Table 2:** Statistically significant multivariate analysis results, showing SMQs with positive disproportionality signals across geriatric age subgroups and CDK4/6 inhibitor subtypes. (The complete results of the univariate and multivariate analyses are provided in Table S5–S7.)

	Abemaciclib		Palbociclib		Ribociclib	
	Adverse Event (SMQ)	OR (95 % CI), *P* value	Adverse Event (SMQ)	OR (95 % CI), *P* value	Adverse Event (SMQ)	OR (95 % CI), *P* value
**65**–**74 years**	Acute renal failure	2.29 [1.63–3.21], P < 0.0001	Dementia	6.84 [3.07–18.15], P < 0.0001	Acute renal failure	2.74 [2.01–3.75], P < 0.0001
	Interstitial lung disease	1.99 [1.46–2.71], P < 0.0001	Hearing and vestibular disorders	2.64 [2.10–3.34], P < 0.0001	Chronic kidney disease	2.50 [1.52–4.13], P = 0.0003
	Embolic and thrombotic events	1.64 [1.21–2.23], P = 0.001	Lens disorders	2.40 [1.74–3.35], P < 0.0001	Cardiac arrhythmias	2.09 [1.65–2.66], P < 0.0001
	Gastrointestinal nonspecific symptoms and therapeutic procedures	0.82 [0.72–0.94], P = 0.004	Hemorrhagic central nervous system vascular conditions	2.26 [1.72–3.00], P < 0.0001	Torsade de pointes/QT prolongation	1.71 [1.23–2.36], P = 0.001
	Hematopoietic cytopenias affecting more than one type of blood cell	0.59 [0.44–0.79], P = 0.0006	Arthritis	2.04 [1.58–2.63], P < 0.0001	Ischemic heart disease	1.67 [1.06–2.60], P = 0.025
			Embolic and thrombotic events vessel type unspecified and mixed arterial and venous	1.68 [1.41–2.00], P < 0.0001	Shock	1.58 [1.21–2.06], P = 0.0008
			Oropharyngeal disorders	0.83 [0.76–0.90], P < 0.0001	Hepatic disorders	0.82 [0.69–0.97], P = 0.019
			Gastrointestinal nonspecific symptoms and therapeutic procedures	0.81 [0.76–0.86], P < 0.0001		
**75**–**84 years**	Dehydration	2.39 [1.60–3.52], P < 0.0001	Dementia	18.78 [8.77–48.79], P < 0.0001	Chronic kidney disease	3.46 [1.98–5.94], P < 0.0001
	Interstitial lung disease	2.38 [1.64–3.42], P < 0.0001	Hearing and vestibular disorders	4.76 [3.81–6.00], P < 0.0001	Acute renal failure	3.26 [2.27–4.63], P < 0.0001
	Acute renal failure	2.06 [1.33–3.13], P = 0.0010	Lens disorders	3.22 [2.31–4.54], P < 0.0001	Cardiac arrhythmias	2.65 [2.00–3.48], P < 0.0001
	Eosinophilic pneumonia	2.04 [1.04–3.80], P = 0.030	Hemorrhagic central nervous system vascular conditions	3.05 [2.30–4.08], P < 0.0001	Torsade de pointes/QT prolongation	1.95 [1.32–2.84], P = 0.0006
	Gastrointestinal nonspecific symptoms and therapeutic procedures	0.76 [0.63–0.91], P = 0.003	Arthritis	2.07 [1.56–2.74], P < 0.0001	Shock	1.63 [1.16–2.25], P = 0.004
	Taste and smell disorders	0.48 [0.21–0.93], P = 0.048	Embolic and thrombotic events vessel type unspecified and mixed arterial and venous	1.86 [1.53–2.25], P < 0.0001	Biliary disorders	0.55 [0.31–0.90], P = 0.027
	Hematopoietic cytopenias affecting more than one type of blood cell	0.32 [0.18–0.51], P < 0.0001	Hematopoietic cytopenias	0.83 [0.78–0.88], P < 0.0001	Hepatic disorders	0.52 [0.40–0.66], P < 0.0001
			Oropharyngeal disorders	0.74 [0.67–0.82], P < 0.0001	Gastrointestinal nonspecific dysfunction	0.45 [0.21–0.84], P = 0.022
			Gastrointestinal nonspecific symptoms and therapeutic procedures	0.69 [0.64–0.74], P < 0.0001		
			Gastrointestinal nonspecific dysfunction	0.67 [0.53–0.84], P = 0.0008		
**85+ years**	Interstitial lung disease	2.16 [0.98–4.23], P = 0.037	Dementia	45.98 [20.69–122.03], P < 0.0001	Acute renal failure	6.21 [3.35–10.83], P < 0.0001
	Gastrointestinal nonspecific symptoms and therapeutic procedures	0.70 [0.49–0.99], P = 0.047	Hearing and vestibular disorders	10.31 [7.94–13.40], P < 0.0001	Chronic kidney disease	5.25 [1.77–12.66], P = 0.0008
	Noninfectious diarrhea	0.62 [0.41–0.90], P = 0.014	Hemorrhagic central nervous system vascular conditions	3.77 [2.49–5.57], P < 0.0001	Ischemic heart disease	3.82 [1.44–8.43], P = 0.002
	Hematopoietic cytopenias affecting more than one type of blood cell	0.09 [0.00–0.41], P = 0.018	Arthritis	2.05 [1.27–3.18], P = 0.002	Cardiac arrhythmias	2.40 [1.23–4.27], P = 0.005
			Embolic and thrombotic events vessel type unspecified and mixed arterial and venous	1.57 [1.10–2.17], P = 0.009	Shock	2.39 [1.19–4.34], P = 0.008
			Gastrointestinal nonspecific symptoms and therapeutic procedures	0.66 [0.58–0.76], P < 0.0001	Hepatic disorders	0.50 [0.25–0.90], P = 0.032
			Hematopoietic cytopenias	0.64 [0.57–0.72], P < 0.0001		
			Gastrointestinal nonspecific dysfunction	0.54 [0.32–0.84], P = 0.011		
			Oropharyngeal disorders	0.53 [0.42–0.64], P < 0.0001		

CI: Confidence interval; OR: Odds ratio; SMQ: Standardised MedDRA Queries.

In palbociclib group, multivariate analysis revealed a progressive increase in the odds of reporting dementia with advancing age, peaking in ≥85-year group (OR = 45.98 [20.69–122.03]; p < 0.0001). Hearing and vestibular disorders demonstrated a stepwise increase, with the most pronounced association in ≥85-year group (OR = 10.31 [7.94–13.40]; p < 0.0001). A comparable age-related trend was also noted for hemorrhagic central nervous system vascular conditions, with the strongest association in ≥85-year group (OR = 3.77 [95 % CI 2.49–5.57]; p < 0.0001). The odds of reporting lens disorders were significantly higher in patients aged 65–74 years (OR = 2.40 [95 % CI 1.74–3.35]; p < 0.0001) and 75–84 years (OR = 3.22 [95 % CI 2.31–4.54]; p < 0.0001). Arthritis was more frequently reported in all geriatric age subgroups, with twofold higher odds in patients aged 65–74 years (OR = 2.04 [95 % CI 1.58–2.63]; p < 0.0001), 75–84 years (OR = 2.07 [95 % CI 1.56–2.74]; p < 0.0001), and ≥85 years (OR = 2.05 [95 % CI 1.27–3.18]; p = 0.002). All geriatric age subgroups showed increased odds of reporting thromboembolic events, with the highest odds in patients aged 75–84 year (OR = 1.86 [95 % CI 1.53–2.25]; p < 0.0001). In contrast, the odds of reporting oropharyngeal disorders declined with advancing age, reaching the lowest level in ≥85-year group (OR = 0.53, [95 % CI 0.42–0.64]; p < 0.0001). Similarly, all geriatric age subgroups showed lower odds of reporting “gastrointestinal nonspecific symptoms and therapeutic procedures”, with an age-related decline and the lowest odds in ≥85-year group (OR = 0.66 [95 % CI 0.58–0.76]; p < 0.0001). Hematopoietic cytopenias were reported less frequently in patients aged 75–84 years (OR = 0.83 [95 % CI 0.78–0.88]; p < 0.0001) and ≥85 years (OR = 0.64 [95 % CI 0.57–0.72]; p < 0.0001).

For ribociclib, advancing age was associated with higher odds of reporting acute renal failure and chronic kidney disease, with the strongest associations in ≥85-year group (OR = 6.21 [95 % CI 3.35–10.83], p < 0.0001 for acute renal failure; OR = 5.25 [95 % CI 1.77–12.66], p = 0.0008 for chronic kidney disease). Cardiac arrhythmias were more frequently reported in all geriatric age subgroups, with the highest odds of reporting in patients aged 75–84 years (OR = 2.65 [95 % CI 2.00–3.48]; p < 0.0001). Significantly elevated odds of reporting Torsade de pointes/QT prolongation were observed in patients aged 65–74 years (OR = 1.71 [95 % CI 1.23–2.36]; p = 0.001) and in those aged 75–84 years (OR = 1.95 [95 % CI 1.32–2.84]; p = 0.0006). The odds of reporting ischemic heart disease were increased in patients aged 65–74 years (OR = 1.67 [95 % CI 1.06–2.60]; p = 0.025) and ≥85 years (OR = 3.82 [95 % CI 1.44–8.43]; p = 0.002). A progressive age-related increase in the odds of reporting shock was observed, peaking in ≥85-year group (OR = 2.39 [95 % CI 1.19–4.34]; p = 0.008). Conversely, hepatic disorders showed significantly reduced odds of reporting in all geriatric age subgroups, with the most pronounced decrease in ≥85-year group (OR = 0.50 [95 % CI 0.25–0.90]; p = 0.032).

## 4 Discussion

In this comprehensive real-world pharmacovigilance analysis, we identified distinct age- and drug-specific toxicity patterns of CDK4/6is, with older adults experiencing disproportionately higher renal, pulmonary, cardiac, and neurocognitive adverse events. Specifically, abemaciclib was predominantly associated with renal and pulmonary complications, palbociclib with neurological and thrombotic toxicities, and ribociclib with renal and cardiac adverse events in older adults.

Age-related alterations in the pharmacokinetics of CDK4/6is may influence the incidence and severity of toxicities [13,14]. Given that rabeprazole-induced gastric acid suppression significantly reduces palbociclib exposure, age-related declines in gastric acid secretion may similarly impair palbociclib bioavailability [15]. Conversely, the age-related decline in liver volume and perfusion may diminish hepatic first-pass clearance, potentially increasing systemic exposure to CDK4/6is [13,14,16]. CDK4/6is are substrates of the P-glycoprotein, whose tissue-specific expression has been shown to vary with age, potentially altering drug bioavailability in older adults [17,18]. Because palbociclib and ribociclib have a large volume of distribution, age-related shifts in adipose tissue and lean mass may also influence their circulating concentrations [13,19]. Additionally, certain geriatric risk factors—such as malnutrition and polypharmacy—may further influence the toxicity profiles. As CDK4/6is are predominantly bound to plasma proteins and the global prevalence of malnutrition among older adults is high, the free fraction of CDK4/6is may be elevated in this vulnerable population [13,20]. Pharmacokinetic interactions with concomitant agents, particularly CYP3A4 inhibitors, may also potentiate CDK4/6i toxicity through impaired clearance [14]. Since multiple age-related factors influence the serum concentrations of CDK4/6is, the combined effects of these factors may lead to heterogeneous toxicity profiles across geriatric age subgroups.

The most striking finding in our study is the progressive rise in dementia reporting with advancing age among patients receiving palbociclib. Consistent with our findings, Prevost et al. identified a neurocognitive safety signal linked to palbociclib in a large-scale disproportionality analysis of the VigiBase database [21]. In a combined analysis of FAERS and VigiBase, Takeda et al. found that palbociclib was more frequently linked to psychiatric and nervous system adverse events than abemaciclib [22]. However, neither study assessed whether neurocognitive adverse events varied across different age groups. While both palbociclib and abemaciclib are lipophilic and capable of crossing the blood–brain barrier, the selective reporting of neurocognitive adverse events with palbociclib may reflect unique off-target pharmacological effects [14]. It has been hypothesized that palbociclib's off-target inhibition of tropomyosin receptor kinases (TRKs), which mediate neuronal development, differentiation, and survival through binding with brain-derived neurotrophic factor, might explain this finding [21]. However, there is no evidence confirming that palbociclib directly interacts with or inhibits TRKs [23]. The potential link between palbociclib and dementia warrants further investigation.

Among palbociclib-treated patients, reports of hearing and vestibular disorders, as well as lens disorders, demonstrated an age-related increase. To our knowledge, this is the first documentation of a potential association between palbociclib and hearing and vestibular disorders, for which the mechanism is currently unknown. Despite the absence of robust evidence from RCTs, a potential association between palbociclib and cataract formation or lens degeneration may be hypothesized, given the documented expression of CDK4 and CDK6 in human lens epithelial cells [24]. The selective reporting of hearing and lens disorders with palbociclib, rather than with other CDK4/6is, merits careful consideration for palbociclib use in older patients with baseline hearing impairment or cataract. Our findings further suggest that palbociclib may be associated with higher rates of thromboembolic events and central nervous system hemorrhages in patients aged ≥65 years. Although the pooled safety analysis of PALOMA-1, -2, and -3 trials reported no central nervous system hemorrhages, a phase II study with 42 participants documented two cases, one of which was a fatal treatment-related subarachnoid hemorrhage in a 73-year-old patient [25,26]. Despite preclinical evidence of the antiarthritic activity of palbociclib in a collagen-induced arthritis model, our study found that arthritis was more frequently reported in patients aged ≥65 years, particularly those receiving concomitant endocrine therapy, likely reflecting the higher baseline prevalence of joint disease and potential additive effects of endocrine therapy [27].

In ribociclib-treated patients, the progressive increase in the odds of renal and cardiac adverse events with advancing age represents a major concern. A recent meta-analysis demonstrated a significant increase in all-grade nephrotoxic adverse events with CDK4/6is [28], and ribociclib may predispose older adults to more frequent and severe renal toxicity due to the high prevalence of polypharmacy. A case of grade 3 acute kidney injury (AKI) associated with ribociclib, resulting in treatment discontinuation, was documented in a 79-year-old patient [29]. Gupta et al. reported six cases of CDK4/6i-associated AKI requiring renal biopsy, four of which were in patients over 75 years [30]. Paradoxically, preclinical studies indicate that targeted inhibition of the CDK4/6 pathway with ribociclib may exert nephroprotective effects by mitigating aberrant cell-cycle activation during cisplatin-induced AKI [31,32]. However, Zhou et al. recently showed that prolonged palbociclib exposure during either acute or post-acute phase of AKI may exacerbate subsequent renal fibrosis, suggesting that transient exposure to CDK4/6is may offer nephroprotection, whereas sustained exposure may promote kidney injury [33].

Cardiovascular toxicity of ribociclib, particularly QT interval prolongation, is well documented [34]. The pooled data from the MONALEESA-2, -3, and -7 trials demonstrated an age-related increase in the incidence of QT prolongation, with rates of 9 % in patients <65 years, 11 % in those aged 65–74 years, and 16 % in patients aged ≥75 years [35]. Ribociclib is also associated with an increased risk of atrial fibrillation and venous thromboembolism [36]. In our study, cardiac arrhythmias were more frequently reported in ribociclib-treated older patients, whereas no age-related differences in venous thromboembolism were observed.

Among patients receiving abemaciclib, the most pronounced age-related differences were noted in renal and pulmonary adverse events in our study. While abemaciclib shares mechanistic similarities with ribociclib in the development of renal toxicity, its propensity to cause diarrhea may result in hypovolemia, further predisposing patients to renal dysfunction [37]. ILD was also more frequently reported in older patients. Despite the exact mechanism remaining unclear, it has been proposed that broader kinase inhibition, combined with high lipophilicity and continuous administration schedule, may contribute to abemaciclib's accumulation in lung tissue [38]. Close follow-up for ILD is critical in older patients receiving abemaciclib, because advanced age has been linked to worse clinical outcomes [39].

Contrary to prior reports that documented higher incidences of hematologic, gastrointestinal, and hepatic toxicities associated with CDK4/6is in older patients [11], our analysis revealed lower reporting frequencies. This may in part be attributable to underreporting in older patients, particularly regarding low-grade toxicities. Preemptive dose reduction in frail older patients may also contribute to lower frequencies. This observation also reflects the important limitations inherent to analyses based on FAERS data. FAERS is subject to underreporting and reporting bias, therefore causality cannot be established. Moreover, the absence of denominator data prevents calculation of true incidence rates, and missing information on patient comorbidities – including renal, hepatic, and cardiac function, frailty, performance status – as well as drug dosing and toxicity grading further limits the ability to accurately assess adverse event risk. It should also be noted that disproportionality analysis detects signals reflecting the odds of reporting, not the true magnitude of risk. Accordingly, our findings should be interpreted with caution, as they do not imply causality but rather serve as hypothesis-generating observations. On the other hand, an important strength of this study is its reliance on a large, heterogeneous cohort that enhances the generalizability of findings. Restricting the analysis to the “primary suspect drug” enhances robustness. However, this approach may lead to underestimation of reporting frequencies and still carries a risk of misattribution of adverse events in patients receiving multiple concomitant medications. The use of age-stratified multivariate analyses further refines interpretability, enabling the identification of subtle age-related patterns in adverse event reporting that might otherwise remain unrecognized. Future prospective studies in older patients with breast cancer are essential to validate these findings and determine their clinical significance. In parallel, mechanistic research should clarify the biological basis of age-related toxicities, supporting personalized treatment approaches.

## CRediT authorship contribution statement

**Bahadır Köylü:** Writing – review & editing, Writing – original draft, Validation, Methodology, Conceptualization. **Buğra Han Esen:** Writing – review & editing, Writing – original draft, Software, Methodology, Formal analysis. **Cevat İlteriş Kıkılı:** Writing – review & editing, Investigation. **Fatih Kemik:** Writing – review & editing, Investigation. **Nazan Demir:** Writing – review & editing, Investigation. **Şeyda Gündüz:** Writing – review & editing, Visualization. **Şahin Laçin:** Writing – review & editing, Visualization. **Deniz Tural:** Writing – review & editing, Visualization. **Didem Tunalı:** Writing – review & editing, Investigation. **Gülistan Bahat:** Writing – review & editing, Visualization, Supervision. **Fatih Selçukbiricik:** Writing – review & editing, Visualization, Supervision, Project administration, Methodology, Conceptualization.

## Data sharing statement

All data used in our study are publicly available at https://fis.fda.gov/extensions/FPD-QDE-FAERS/FPD-QDE-FAERS.html.

## Trial registration

Not applicable.

## Funding statement

This work received no specific grant from any funding agency in the public, commercial, or not-for-profit sectors.

## Declaration of competing interest

The authors declare that they have no known competing financial interests or personal relationships that could have appeared to influence the work reported in this paper.
