# Supplementary material for: Age-stratified toxicity patterns of CDK4/6 inhibitors in older women with breast cancer: Disproportionality analysis from the FAERS database
Source: Breast. 2025 Dec 29;85:104687. doi: 10.1016/j.breast.2025.104687 (PMC12813243; doi:10.1016/j.breast.2025.104687)

**Supplementary Appendix**

**Table of Contents**

- **Supplementary Table S1**. List of chemotherapies with the international nonproprietary names (INNs)
- **Supplementary Table S2**. List of other targeted therapies with the international nonproprietary names (INNs)
- **Supplementary Table S3**. List of endocrine therapies with the international nonproprietary names (INNs)
- **Supplementary Table S4.** Summary of reporting odds ratio (ROR) used for signal detection
- **Supplementary Table S5.** Univariate and multivariate analysis of SMQs with positive disproportionality signals in ≥60 abemaciclib-associated cases
- **Supplementary Table S6.** Univariate and multivariate analysis of SMQs with positive disproportionality signals in ≥60 palbociclib-associated cases
- **Supplementary Table S7.** Univariate and multivariate analysis of SMQs with positive disproportionality signals in ≥60 ribociclib-associated cases
- **Supplementary Figure S1.** Disproportionality Analysis of Standardised MedDRA Queries (SMQs) for CDK4/6 Inhibitors (Palbociclib, Ribociclib, Abemaciclib) Using Reporting Odds Ratios (RORs)

**Supplementary Table S1. List of chemotherapies with the international nonproprietary names (INNs)**

| Aclarubicin, Altretamine, Amrubicin, Amsacrine, Asparaginase, Azacitidine, Belotecan, Bendamustine, Bleomycin, Busulfan, Cabazitaxel, Capecitabine, Carboplatin, Carboquone, Carmofur, Carmustine, Chlorambucil, Cisplatin, Cladribine, Clofarabine, Cyclophosphamide, Cytarabine, Dacarbazine, Dactinomycin, Daunorubicin, Decitabine, Demecolcine, Docetaxel, Doxorubicin, Epirubicin, Eribulin, Estramustine, Etoglucid, Etirinotecan pegol, Etoposide, Fludarabine, Floxuridine, Fluorouracil, Fotemustine, Gemcitabine, Hydroxycarbamide, Idarubicin, Ifosfamide, Irinotecan, Ixabepilone, Lomustine, Lonidamine, Lurbinectedin, Mannosulfan, Masoprocol, Melphalan, Melphalan flufenamide, Mercaptopurine, Methotrexate, Mitomycin, Mitotane, Mitoxantrone, Mitobronitol, Mitoguazone, Nelarabine, Nimustine, Oxaliplatin, Paclitaxel, Paclitaxel and encequidar, Paclitaxel poliglumex, Pegaspargase, Pemetrex, Pentostatin, Pipobroman, Pirarubicin, Pixantrone, Plicamycin, Polyplatillen, Pralatrexate, Prednimustine, Procarbazine, Raltitrexed, Ranimustine, Satraplatin, Semustine, Streptozocin, Tegafur, Temozolomide, Teniposide, Thiotepa, Tiazofurine, Tioguanine, Topotecan, Trabectedin, Treosulfan, Triaziquone, Trofosfamide, Uramustine, Valrubicin, Vinblastine, Vincristine, Vindesine, Vinflunine, Vintafolide, Vinorelbine, Vosaroxin, Zorubicin |
| --- |

**Supplementary Table S2. List of other targeted therapies with the international nonproprietary names (INNs)**

| Acalabrutinib, Adagrasib, Aflibercept, Alectinib, Alpelisib, Amivantamab, Anagrelide, Asciminib, Asimomib, Arsenic trioxide, Atezolizumab, Aumolertinib, Avapritinib, Axitinib, Belantamab, Belantamab mafodotin, Belzutifan, Bendamustine, Bevacizumab, Binimetinib, Blinatumomab, Bortezomib, Bosutinib, Brigatinib, Brentuximab, Brentuximab vedotin, Cabozantinib, Capivasertib, Capmatinib, Carfilzomib, Catumaxomab, Cediranib, Cetuximab, Cobimetinib, Copanlisib, Crizotinib, Dacomitinib, Dabrafenib, Daratumumab, Dasatinib, Denileukin, Denileukin diftitox, Duvelisib, Edrecolomab, Elotuzumab, Enasidenib, Encorafenib, Entinostat, Entrectinib, Enfortumab, Enfortumab vedotin, Epacadostat, Erdafitinib, Erlotinib, Everolimus, Fedratinib, Fruquintinib, Futibatinib, Gefitinib, Gilteritinib, Glasdegib, Gemtuzumab, Gemtuzumab ozogamicin, Ibrutinib, Idelalisib, Icotinib, Imatinib, Infigratinib, Inotuzumab, Inotuzumab ozogamicin, Isatuximab, Ivosidenib, Ixazomib, Ixabepilone, Lazertinib, Larotrectinib, Lapatinib, Lenvatinib, Loncastuximab, Loncastuximab tesirine, Lorlatinib, Masitinib, Midostaurin, Mobocertinib, Margetuximab, Mogamulizumab, Momelotinib, Moxetumomab, Moxetumomab pasudotox, Naxitamab, Necitumumab, Neratinib, Nilotinib, Nintedanib, Niraparib, Obinutuzumab, Oblimersen, Ofatumumab, Olaparib, Olmutinib, Omacetaxine, Omacetaxine mepesuccinate, Oportuzumab, Oportuzumab monatox, Osimertinib, Orelabrutinib, Pacritinib, Panitumumab, Panobinostat, Parsaclisib, Pazopanib, Pamiparib, Parsaclisib, Pemetrexed, Pemigatinib, Pertuzumab, Pexidartinib, Pirarubicin, Pirtobrutinib, Plitidepsin, Polatuzumab, Polatuzumab vedotin, Ponatinib, Pralsetinib, Ramucirumab, Regorafenib, Ridaforolimus, Ripretinib, Rituximab, Rociletinib, Romidepsin, Ruxolitinib, Sacituzumab, Sacituzumab govitecan, Sabatolimab, Selinexor, Selpercatinib, Selumetinib, Sirolimus, Sitravatinib, Sonidegib, Sorafenib, Sotorasib, Sunitinib, Surufatinib, Talazoparib, Tafasitamab, Tagraxofusp, Tazemetostat, Tebentafusp, Tepotinib, Temsirolimus, Tisotumab, Tisotumab vedotin, Tivozanib, Trametinib, Trastuzumab, Trastuzumab deruxtecan, Trastuzumab duocarmazine, Trastuzumab emtansine, Tucatinib, Umbralisib, Vandetanib, Veliparib, Venetoclax, Vemurafenib, Visomdegib, Vismodegib, Voprotomab, Vorinostat, Zanubrutinib, Zorubicin. |
| --- |

**Supplementary Table S3. List of other endocrine therapies with the international nonproprietary names (INNs)**

| Aminoglutethimide, Anastrozole, Elacestrant, Exemestane, Formestane, Fulvestrant, Letrozole, Tamoxifen, Toremifene, Vorozole. |
| --- |

**Supplementary Table S4. Summary of reporting odds ratio (ROR) used for signal detection**

|  | **ICIs** | **All Other Drugs** | **Total** |
| --- | --- | --- | --- |
| Adverse event | *a* | *c* | *a+c* |
| All other adverse events | *b* | *d* | *b+d* |
| Total | *a+b* | *c+d* | *a+b+c+d* |

a = The number of reports of ICIs with the adverse event of interest (P).

b = The number of reports of ICIs with all other adverse events.

c = The number of reports of all other drugs with the adverse event of interest (P).

d = The number of reports of all other drugs with all other adverse events

| **Algorithm** | **Formula** | **Criteria** |
| --- | --- | --- |
| ROR | ROR = $\frac{\frac{a}{b}}{\frac{c}{d}}$  95%CI = $e^{lnln \left( ROR \right) \pm1.96\sqrt{\frac{1}{a}+\frac{1}{b}+\frac{1}{c}+\frac{1}{d}}}$ | ROR_025_ > 1  N≥3 |

Abbreviations: ROR, reporting odds ratio; CI, confidence interval; ROR025, the lower limit of the 95% two-sided CI of the ROR; N, the number of co-occurrences

**Supplementary Table S5. Univariate and multivariate analysis of SMQs with positive disproportionality signals in ≥60 abemaciclib-associated cases**

|  | **Univariate analysis** | | **Multivariate analysis** | |
| --- | --- | --- | --- | --- |
|  | **OR (95% CI)** | ***P* value** | **OR (95% CI)** | ***P* value** |
| **Gastrointestinal nonspecific symptoms and therapeutic procedures (n=2,050, 41.7%);**  *Diarrhoea, Nausea, Vomiting* | | | | |
| Age subgroup (Ref: <65 years)  · 65-74 years  · 75-84 years  · +85 years | 0.78 [0.68-0.89]  0.72 [0.60-0.86]  0.69 [0.48-0.98] | 0.0003  0.0003  0.043 | 0.82 [0.72-0.94]  0.76 [0.63-0.91]  0.70 [0.49-0.99] | 0.004  0.003  0.047 |
| Additional therapies, previous/concomitant  · Endocrine Therapy  · Other Targeted Therapy  · Chemotherapy | 0.59 [0.52-0.66]  0.70 [0.36-1.28]  0.61 [0.28-1.24] | <0.0001  0.26  0.19 | 0.60 [0.53-0.68] | <0.0001 |
| **Noninfectious diarrhoea (n=1,697, 34.5%);**  *Diarrhoea, Frequent bowel movements, Defaecation urgency* | | | | |
| Age subgroup (Ref: <65 years)  · 65-74 years  · 75-84 years  · +85 years | 0.89 [0.78-1.02]  0.80 [0.66-0.96]  0.61 [0.41-0.89] | 0.10  0.017  0.013 | 0.83 [0.69-1.00]  0.62 [0.41-0.90] | 0.054  0.014 |
| Additional therapies, previous/concomitant  · Endocrine Therapy  · Other Targeted Therapy  · Chemotherapy | 0.66 [0.58-0.76]  0.69 [0.34-1.30]  0.51 [0.20-1.11] | <0.0001  0.27  0.11 | 0.67 [0.59-0.76] | <0.0001 |
| **Hepatic disorders (n=478, 9.7%);**  *Hepatic function abnormal, Drug-induced liver injury, Alanine aminotransferase increased* | | | | |
| Age subgroup (Ref: <65 years)  · 65-74 years  · 75-84 years  · +85 years | 1.10 [0.88-1.36]  0.80 [0.58-1.09]  0.56 [0.25-1.07] | 0.41  0.17  0.11 |  |  |
| Additional therapies, previous/concomitant  · Endocrine Therapy  · Other Targeted Therapy  · Chemotherapy | 3.56 [2.94-4.33]  1.16 [0.40-2.70]  0.93 [0.22-2.62] | <0.0001  0.75  0.90 | 3.63 [2.99-4.42] | <0.0001 |
| **Haematopoietic cytopenias affecting more than one type of blood cell (n=281, 5.7%);**  *Myelosuppression, Full blood count decreased, Pancytopenia* | | | | |
| Age subgroup (Ref: <65 years)  · 65-74 years  · 75-84 years  · +85 years | 0.64 [0.47-0.86]  0.35 [0.20-0.56]  0.09 [0.00-0.42] | 0.004  <0.0001  0.019 | 0.59 [0.44-0.79]  0.32 [0.18-0.51]  0.09 [0.00-0.41] | 0.0006  <0.0001  0.018 |
| Additional therapies, previous/concomitant  · Endocrine Therapy  · Other Targeted Therapy  · Chemotherapy | 1.92 [1.50-2.44]  0.77 [0.12-2.50]  1.66 [0.40-4.69] | <0.0001  0.71  0.41 | 2.07 [1.62-2.64] | <0.0001 |
| **Interstitial lung disease (n=233, 4.7%);**  *Interstitial lung disease, Pneumonitis, Pulmonary fibrosis* | | | | |
| Age subgroup (Ref: <65 years)  · 65-74 years  · 75-84 years  · +85 years | 2.33 [1.72-3.16]  2.79 [1.94-3.98]  2.19 [1.01-4.22] | <0.0001  <0.0001  0.030 | 1.99 [1.46-2.71]  2.38 [1.64-3.42]  2.16 [0.98-4.23] | <0.0001  <0.0001  0.037 |
| Additional therapies, previous/concomitant  · Endocrine Therapy  · Other Targeted Therapy  · Chemotherapy | 5.54 [4.17-7.46]  3.15 [1.19-6.98]  1.30 [0.21-4.33] | <0.0001  0.010  0.72 | 5.11 [3.83-6.89]  2.39 [0.88-5.54] | <0.0001  0.059 |
| **Embolic and thrombotic events (n=214, 4.4%);**  *Pulmonary embolism, Thrombosis, Deep vein thrombosis* | | | | |
| Age subgroup (Ref: <65 years)  · 65-74 years  · 75-84 years  · +85 years | 1.72 [1.26-2.32]  1.17 [0.74-1.79]  1.63 [0.72-3.21] | 0.0005  0.48  0.20 | 1.64 [1.21-2.23] | 0.001 |
| Additional therapies, previous/concomitant  · Endocrine Therapy  · Other Targeted Therapy  · Chemotherapy | 1.58 [1.19-2.08]  1.02 [0.17-3.35]  1.42 [0.23-4.74] | 0.001  0.98  0.63 | 1.52 [1.15-2.00] | 0.003 |
| **Acute renal failure (n=182, 3.7%);**  *Acute kidney injury, Renal impairment, Renal failure* | | | | |
| Age subgroup (Ref: <65 years)  · 65-74 years  · 75-84 years  · +85 years | 2.40 [1.72-3.36]  2.16 [1.39-3.28]  2.09 [0.86-4.33] | <0.0001  0.0004  0.069 | 2.29 [1.63-3.21]  2.06 [1.33-3.13] | <0.0001  0.0010 |
| Additional therapies, previous/concomitant  · Endocrine Therapy  · Other Targeted Therapy  · Chemotherapy | 1.76 [1.30-2.37]  0.59 [0.03-2.72]  3.65 [1.07-9.39] | 0.0002  0.60  0.016 | 1.59 [1.17-2.15]    3.19 [0.93-8.38] | 0.003    0.034 |
| **Haematopoietic thrombocytopenia (n=182, 3.7%);**  *Platelet count decreased, Thrombocytopenia, Platelet production decreased* | | | | |
| Age subgroup (Ref: <65 years)  · 65-74 years  · 75-84 years  · +85 years | 1.35 [0.96-1.89]  1.43 [0.92-2.15]  0.21 [0.01-0.95] | 0.079  0.10  0.12 |  |  |
| Additional therapies, previous/concomitant  · Endocrine Therapy  · Other Targeted Therapy  · Chemotherapy | 1.93 [1.43-2.59]  1.21 [0.20-3.97]  3.65 [1.07-9.39] | <0.0001  0.79  0.016 | 1.84 [1.36-2.48]    3.09 [0.90-8.04] | <0.0001    0.038 |
| **Dehydration (n=162, 3.3%);**  *Dehydration, Hypovolemia, Hypovolemic shock* | | | | |
| Age subgroup (Ref: <65 years)  · 65-74 years  · 75-84 years  · +85 years | 1.20 [0.81-1.74]  2.39 [1.60-3.52]  0.78 [0.19-2.12] | 0.36  <0.0001  0.68 | 2.39 [1.60-3.52] | <0.0001 |
| Additional therapies, previous/concomitant  · Endocrine Therapy  · Other Targeted Therapy  · Chemotherapy | 1.32 [0.95-1.81]  1.37 [0.22-4.50]  0.00 [0.00-950.02] | 0.094  0.67  0.97 |  |  |
| **Taste and smell disorders (n=112, 2.3%);0**  *Taste disorder, Dysgeusia, Ageusia* | | | | |
| Age subgroup (Ref: <65 years)  · 65-74 years  · 75-84 years  · +85 years | 0.65 [0.39-1.03]  0.48 [0.21-0.93]  1.35 [0.47-3.08] | 0.076  0.048  0.52 | 0.48 [0.21-0.93] | 0.048 |
| Additional therapies, previous/concomitant  · Endocrine Therapy  · Other Targeted Therapy  · Chemotherapy | 0.74 [0.48-1.12]  0.98 [0.06-4.53]  0.00 [0.00-1369.93] | 0.17  0.98  0.98 |  |  |
| **Haematopoietic erythropenia (n=69, 1.4%);** *Red blood cell count decreased, Anemia macrocytic* | | | | |
| Age subgroup (Ref: <65 years)  · 65-74 years  · 75-84 years  · +85 years | 1.09 [0.63-1.84]  0.77 [0.32-1.62]  0.00 [0.00-1340.46] | 0.75  0.53  0.98 |  |  |
| Additional therapies, previous/concomitant  · Endocrine Therapy  · Other Targeted Therapy  · Chemotherapy | 0.67 [0.37-1.13]  3.34 [0.54-11.13]  2.21 [0.12-10.53] | 0.15  0.10  0.44 |  |  |
| **Eosinophilic pneumonia (n=66, 1.3%);**  *Pneumonitis, Eosinophilic pneumonia, Eosinophilic pneumonia chronic* | | | | |
| Age subgroup (Ref: <65 years)  · 65-74 years  · 75-84 years  · +85 years | 1.54 [0.86-2.70]  2.20 [1.13-4.10]  1.38 [0.22-4.64] | 0.14  0.016  0.66 | 2.04 [1.04-3.80] | 0.030 |
| Additional therapies, previous/concomitant  · Endocrine Therapy  · Other Targeted Therapy  · Chemotherapy | 2.07 [1.27-3.38]  0.00 [0.00-144771.64]  0.00 [0.00-356283114.58] | 0.003  0.98  0.98 | 1.96 [1.20-3.20] | 0.007 |

**Supplementary Table S6. Univariate and multivariate analysis of SMQs with positive disproportionality signals in ≥60 palbociclib-associated cases**

|  | **Univariate analysis** | | **Multivariate analysis** | |
| --- | --- | --- | --- | --- |
|  | **OR (95% CI)** | ***P* value** | **OR (95% CI)** | ***P* value** |
| **Haematopoietic cytopenias (n=10,656, 28.0%);**  *White blood cell count decreased, Neutropenia, Platelet count decreased* | | | | |
| Age subgroup (Ref: <65 years)  · 65-74 years  · 75-84 years  · +85 years | 0.97 [0.92-1.02]  0.83 [0.78-0.88]  0.63 [0.56-0.70] | 0.20  <0.0001  <0.0001 | 0.83 [0.78-0.88]  0.64 [0.57-0.72] | <0.0001  <0.0001 |
| Additional therapies, previous/concomitant  · Endocrine Therapy  · Other Targeted Therapy  · Chemotherapy | 1.62 [1.55-1.69]  1.03 [0.82-1.28]  1.20 [0.90-1.57] | <0.0001  0.80  0.21 | 1.61 [1.54-1.69] | <0.0001 |
| **Gastrointestinal nonspecific symptoms and therapeutic procedures (n=7457, 19.6%);**  *Nausea, Diarrhoea, Vomiting* | | | | |
| Age subgroup (Ref: <65 years)  · 65-74 years  · 75-84 years  · +85 years | 0.81 [0.76-0.86]  0.69 [0.64-0.74]  0.66 [0.58-0.75] | <0.0001  <0.0001  <0.0001 | 0.81 [0.76-0.86]  0.69 [0.64-0.74]  0.66 [0.58-0.76] | <0.0001  <0.0001  <0.0001 |
| Additional therapies, previous/concomitant  · Endocrine Therapy  · Other Targeted Therapy  · Chemotherapy | 1.19 [1.13-1.25]  1.32 [1.04-1.66]  1.00 [0.71-1.37] | <0.0001  0.019  0.98 | 1.19 [1.13-1.25]  1.21 [0.95-1.51] | <0.0001  0.12 |
| **Oropharyngeal disorders (n=3,263, 8.6%);**  *Stomatitis, Oropharyngeal pain, Oral pain* | | | | |
| Age subgroup (Ref: <65 years)  · 65-74 years  · 75-84 years  · +85 years | 0.83 [0.76-0.90]  0.74 [0.67-0.81]  0.52 [0.42-0.63] | <0.0001  <0.0001  <0.0001 | 0.83 [0.76-0.90]  0.74 [0.67-0.82]  0.53 [0.42-0.64] | <0.0001  <0.0001  <0.0001 |
| Additional therapies, previous/concomitant  · Endocrine Therapy  · Other Targeted Therapy  · Chemotherapy | 1.25 [1.16-1.34]  1.37 [0.99-1.86]  0.96 [0.58-1.49] | <0.0001  0.046  0.86 | 1.24 [1.15-1.33]  1.24 [0.89-1.68] | <0.0001  0.18 |
| **Haemorrhages (n=1792, 4.7%);**  *Epistaxis, Contusion, Haemorrhage* | | | | |
| Age subgroup (Ref: <65 years)  · 65-74 years  · 75-84 years  · +85 years | 0.99 [0.89-1.11]  1.10 [0.97-1.24]  1.08 [0.86-1.34] | 0.91  0.15  0.51 |  |  |
| Additional therapies, previous/concomitant  · Endocrine Therapy  · Other Targeted Therapy  · Chemotherapy | 1.33 [1.21-1.47]  0.96 [0.58-1.50]  1.11 [0.59-1.91] | <0.0001  0.88  0.72 | 1.34 [1.22-1.47] | <0.0001 |
| **Embolic and thrombotic events vessel type unspecified and mixed arterial and venous**  **(n=758, 2.0%);**  *Thrombosis, Cerebrovascular accident, Embolism* | | | | |
| Age subgroup (Ref: <65 years)  · 65-74 years  · 75-84 years  · +85 years | 1.68 [1.41-2.00]  1.85 [1.53-2.25]  1.55 [1.09-2.14] | <0.0001  <0.0001  0.011 | 1.68 [1.41-2.00]  1.86 [1.53-2.25]  1.57 [1.10-2.17] | <0.0001  <0.0001  0.009 |
| Additional therapies, previous/concomitant  · Endocrine Therapy  · Other Targeted Therapy  · Chemotherapy | 1.23 [1.06-1.42]  0.63 [0.22-1.36]  1.09 [0.39-2.39] | 0.005  0.30  0.85 | 1.23 [1.07-1.42] | 0.004 |
| **Hearing and vestibular disorders (n=706, 1.9%);**  *Hypoacusis, Vertigo, Deafness* | | | | |
| Age subgroup (Ref: <65 years)  · 65-74 years  · 75-84 years  · +85 years | 2.64 [2.10-3.34]  4.76 [3.81-6.00]  10.31 [7.94-13.40] | <0.0001  <0.0001  <0.0001 | 2.64 [2.10-3.34]  4.76 [3.81-6.00]  10.31 [7.94-13.40] | <0.0001  <0.0001  <0.0001 |
| Additional therapies, previous/concomitant  · Endocrine Therapy  · Other Targeted Therapy  · Chemotherapy | 1.04 [0.90-1.21]  0.27 [0.04-0.83]  0.23 [0.01-1.02] | 0.56  0.063  0.14 |  |  |
| **Taste and smell disorders (n=674, 1.8%);**  *Dysgeusia, Taste disorder, Ageusia* | | | | |
| Age subgroup (Ref: <65 years)  · 65-74 years  · 75-84 years  · +85 years | 1.00 [0.83-1.19]  1.06 [0.87-1.29]  0.68 [0.43-1.01] | 0.98  0.56  0.074 |  |  |
| Additional therapies, previous/concomitant  · Endocrine Therapy  · Other Targeted Therapy  · Chemotherapy | 1.33 [1.15-1.55]  1.15 [0.52-2.16]  1.23 [0.44-2.69] | 0.0002  0.70  0.65 | 1.33 [1.14-1.55] | 0.0003 |
| **Gastrointestinal nonspecific dysfunction (n=597, 1.6%);**  *Dyspepsia, Gastrooesophageal reflux disease* | | | | |
| Age subgroup (Ref: <65 years)  · 65-74 years  · 75-84 years  · +85 years | 0.89 [0.74-1.06]  0.67 [0.52-0.84]  0.52 [0.31-0.82] | 0.20  0.0007  0.008 | 0.67 [0.53-0.84]  0.54 [0.32-0.84] | 0.0008  0.011 |
| Additional therapies, previous/concomitant  · Endocrine Therapy  · Other Targeted Therapy  · Chemotherapy | 1.48 [1.25-1.74]  1.64 [0.81-2.92]  0.55 [0.09-1.71] | <0.0001  0.13  0.40 | 1.47 [1.25-1.72] | <0.0001 |
| **Arthritis (n=378, 1.0%);**  *Arthritis, Osteoarthritis, Gout* | | | | |
| Age subgroup (Ref: <65 years)  · 65-74 years  · 75-84 years  · +85 years | 2.04 [1.59-2.63]  2.05 [1.55-2.72]  1.96 [1.21-3.03] | <0.0001  <0.0001  0.004 | 2.04 [1.58-2.63]  2.07 [1.56-2.74]  2.05 [1.27-3.18] | <0.0001  <0.0001  0.002 |
| Additional therapies, previous/concomitant  · Endocrine Therapy  · Other Targeted Therapy  · Chemotherapy | 2.02 [1.64-2.49]  0.76 [0.19-1.99]  0.87 [0.14-2.73] | <0.0001  0.64  0.85 | 2.03 [1.65-2.50] | <0.0001 |
| **Haemorrhagic central nervous system vascular conditions (n=370, 1.0%);**  *Cerebrovascular accident, Cerebral haemorrhage, Subdural haematoma* | | | | |
| Age subgroup (Ref: <65 years)  · 65-74 years  · 75-84 years  · +85 years | 2.26 [1.72-3.00]  3.05 [2.30-4.08]  3.77 [2.49-5.57] | <0.0001  <0.0001  <0.0001 | 2.26 [1.72-3.00]  3.05 [2.30-4.08]  3.77 [2.49-5.57] | <0.0001  <0.0001  <0.0001 |
| Additional therapies, previous/concomitant  · Endocrine Therapy  · Other Targeted Therapy  · Chemotherapy | 0.92 [0.74-1.13]  0.52 [0.09-1.61]  0.00 [0.00-0.00] | 0.42  0.35  0.96 |  |  |
| **Lens disorders (n=258, 0.7%);**  *Cataract, Cataract operation, Cataract subcapsular* | | | | |
| Age subgroup (Ref: <65 years)  · 65-74 years  · 75-84 years  · +85 years | 2.41 [1.75-3.35]  3.19 [2.29-4.50]  1.50 [0.72-2.81] | <0.0001  <0.0001  0.24 | 2.40 [1.74-3.35]  3.22 [2.31-4.54] | <0.0001  <0.0001 |
| Additional therapies, previous/concomitant  · Endocrine Therapy  · Other Targeted Therapy  · Chemotherapy | 2.21 [1.72-2.86]  0.37 [0.02-1.64]  0.00 [0.00-0.00] | <0.0001  0.32  0.96 | 2.22 [1.73-2.87] | <0.0001 |
| **Dementia (n=126, 0.3%);**  *Dementia, Dementia Alzheimer's type, Senile dementia* | | | | |
| Age subgroup (Ref: <65 years)  · 65-74 years  · 75-84 years  · +85 years | 6.83 [3.07-18.13]  18.83 [8.79-48.93]  46.90 [21.11-124.45] | <0.0001  <0.0001<0.0001 | 6.84 [3.07-18.15]  18.78 [8.77-48.79]  45.98 [20.69-122.03] | <0.0001  <0.0001  <0.0001 |
| Additional therapies, previous/concomitant  · Endocrine Therapy  · Other Targeted Therapy  · Chemotherapy | 0.68 [0.46-0.97]  1.54 [0.25-4.85]  0.00 [0.00-3.26] | 0.037  0.55  0.98 | 0.72 [0.49-1.03] | 0.081 |

**Supplementary Table S7. Univariate and multivariate analysis of SMQs with positive disproportionality signals in ≥60 ribociclib-associated cases**

|  | **Univariate analysis** | | **Multivariate analysis** | |
| --- | --- | --- | --- | --- |
|  | **OR (95% CI)** | ***P* value** | **OR (95% CI)** | ***P* value** |
| **Hepatic disorders (n=940, 14.9%);**  *Alanine aminotransferase increased, Aspartate aminotransferase increased, Hepatic enzyme increased* | | | | |
| Age subgroup (Ref: <65 years)  · 65-74 years  · 75-84 years  · +85 years | 0.82 [0.70-0.97]  0.52 [0.40-0.66]  0.47 [0.24-0.84] | 0.022  <0.0001  0.018 | 0.82 [0.69-0.97]  0.52 [0.40-0.66]  0.50 [0.25-0.90] | 0.019  <0.0001  0.032 |
| Additional therapies, previous/concomitant  · Endocrine Therapy  · Other Targeted Therapy  · Chemotherapy | 1.74 [1.51-2.00]  1.50 [0.96-2.26]  2.00 [1.26-3.08] | <0.0001  0.066  0.002 | 1.72 [1.49-1.98]    1.67 [1.05-2.57] | <0.0001    0.025 |
| **Haemodynamic oedema effusions and fluid overload (n=706, 14.4%);**  *Pleural effusion, Peripheral swelling, Pulmonary oedema* | | | | |
| Age subgroup (Ref: <65 years)  · 65-74 years  · 75-84 years  · +85 years | 0.88 [0.72-1.06]  1.02 [0.80-1.28]  0.51 [0.23-0.98] | 0.18  0.87  0.066 |  |  |
| Additional therapies, previous/concomitant  · Endocrine Therapy  · Other Targeted Therapy  · Chemotherapy | 1.27 [1.09-1.49]  1.44 [0.86-2.28]  1.89 [1.12-3.03] | 0.002  0.14  0.011 | 1.25 [1.07-1.47]    1.76 [1.04-2.83] | 0.005    0.026 |
| **Cardiac arrhythmias (n=383, 7.8%);**  *Electrocardiogram QT prolonged, Arrhythmia, Atrial fibrillation* | | | | |
| Age subgroup (Ref: <65 years)  · 65-74 years  · 75-84 years  · +85 years | 2.09 [1.65-2.66]  2.65 [2.00-3.48]  2.40 [1.23-4.27] | <0.0001  <0.0001  0.005 | 2.09 [1.65-2.66]  2.65 [2.00-3.48]  2.40 [1.23-4.27] | <0.0001  <0.0001  0.005 |
| Additional therapies, previous/concomitant  · Endocrine Therapy  · Other Targeted Therapy  · Chemotherapy | 1.05 [0.86-1.30]  0.00 [0.00-0.00]  0.45 [0.11-1.20] | 0.61  0.97  0.17 |  |  |
| **Shock (n=296, 6.0%);**  *Electrocardiogram QT prolonged, Cardiac arrest, Cardio-respiratory arrest* | | | | |
| Age subgroup (Ref: <65 years)  · 65-74 years  · 75-84 years  · +85 years | 1.58 [1.21-2.06]  1.63 [1.16-2.25]  2.39 [1.19-4.34] | 0.0008  0.004  0.008 | 1.58 [1.21-2.06]  1.63 [1.16-2.25]  2.39 [1.19-4.34] | 0.0008  0.004  0.008 |
| Additional therapies, previous/concomitant  · Endocrine Therapy  · Other Targeted Therapy  · Chemotherapy | 1.01 [0.80-1.28]  0.00 [0.00-0.00]  0.39 [0.06-1.24] | 0.93  0.97  0.19 |  |  |
| **Acute renal failure (n=235, 4.8%);**  *Acute kidney injury, Renal impairment, Renal failure* | | | | |
| Age subgroup (Ref: <65 years)  · 65-74 years  · 75-84 years  · +85 years | 2.74 [2.01-3.75]  3.26 [2.27-4.63]  6.21 [3.35-10.83] | <0.0001  <0.0001  <0.0001 | 2.74 [2.01-3.75]  3.26 [2.27-4.63]  6.21 [3.35-10.83] | <0.0001  <0.0001  <0.0001 |
| Additional therapies, previous/concomitant  · Endocrine Therapy  · Other Targeted Therapy  · Chemotherapy | 1.27 [0.98-1.65]  1.02 [0.36-2.28]  0.24 [0.01-1.10] | 0.074  0.96  0.16 |  |  |
| **Depression and suicide/self injury (n=212, 4.3%);**  *Depression, Depressed mood, Discouragement* | | | | |
| Age subgroup (Ref: <65 years)  · 65-74 years  · 75-84 years  · +85 years | 0.91 [0.65-1.25]  0.96 [0.62-1.44]  0.44 [0.07-1.40] | 0.57  0.86  0.25 |  |  |
| Additional therapies, previous/concomitant  · Endocrine Therapy  · Other Targeted Therapy  · Chemotherapy | 2.63 [1.96-3.55]  0.90 [0.28-2.17]  1.14 [0.35-2.75] | <0.0001  0.84  0.80 | 2.62 [1.96-3.54] | <0.0001 |
| **Torsade de pointes/QT prolongation (n=201, 4.1%);**  *Electrocardiogram QT prolonged, Electrocardiogram QT interval abnormal, Long QT syndrome* | | | | |
| Age subgroup (Ref: <65 years)  · 65-74 years  · 75-84 years  · +85 years | 1.71 [1.23-2.36]  1.95 [1.32-2.84]  1.28 [0.39-3.12] | 0.001  0.0006  0.64 | 1.71 [1.23-2.36]  1.95 [1.32-2.84] | 0.001  0.0006 |
| Additional therapies, previous/concomitant  · Endocrine Therapy  · Other Targeted Therapy  · Chemotherapy | 1.14 [0.86-1.51]  0.00 [0.00-0.00]  0.29 [0.02-1.30] | 0.35  0.97  0.22 |  |  |
| **Biliary disorders (n=194, 3.9%);**  *Blood bilirubin increased, Jaundice, Cholelithiasis* | | | | |
| Age subgroup (Ref: <65 years)  · 65-74 years  · 75-84 years  · +85 years | 0.88 [0.63-1.23]  0.55 [0.31-0.90]  0.00 [0.00-0.00] | 0.47  0.026  0.97 | 0.55 [0.31-0.90] | 0.027 |
| Additional therapies, previous/concomitant  · Endocrine Therapy  · Other Targeted Therapy  · Chemotherapy | 1.95 [1.45-2.63]  1.26 [0.44-2.80]  1.25 [0.38-3.03] | <0.0001  0.62  0.66 | 1.93 [1.44-2.60] | <0.0001 |
| **Gastrointestinal nonspecific dysfunction (n=131, 2.7%);**  *Dyspepsia, Gastrooesophageal reflux disease* | | | | |
| Age subgroup (Ref: <65 years)  · 65-74 years  · 75-84 years  · +85 years | 0.73 [0.47-1.10]  0.44 [0.20-0.83]  0.64 [0.10-2.04] | 0.14  0.019  0.53 | 0.45 [0.21-0.84] | 0.022 |
| Additional therapies, previous/concomitant  · Endocrine Therapy  · Other Targeted Therapy  · Chemotherapy | 1.57 [1.11-2.24]  1.11 [0.27-2.98]  2.94 [1.13-6.30] | 0.012  0.86  0.012 | 1.52 [1.07-2.18]    2.52 [0.96-5.43] | 0.019    0.033 |
| **Gastrointestinal nonspecific inflammation (n=119, 2.4%);**  *Gastritis, Colitis, Reflux gastritis* | | | | |
| Age subgroup (Ref: <65 years)  · 65-74 years  · 75-84 years  · +85 years | 1.14 [0.74-1.71]  0.97 [0.54-1.66]  0.42 [0.02-1.91] | 0.55  0.93  0.39 |  |  |
| Additional therapies, previous/concomitant  · Endocrine Therapy  · Other Targeted Therapy  · Chemotherapy | 3.12 [2.10-4.76]  1.22 [0.30-3.30]  3.26 [1.25-7.00] | <0.0001  0.73  0.006 | 3.02 [2.03-4.62]    2.50 [0.95-5.40] | <0.0001    0.035 |
| **Ischaemic heart disease (n=104, 2.1%);**  *Myocardial infarction, Acute myocardial infarction, Angina pectoris* | | | | |
| Age subgroup (Ref: <65 years)  · 65-74 years  · 75-84 years  · +85 years | 1.67 [1.06-2.60]  1.62 [0.90-2.78]  3.82 [1.44-8.43] | 0.025  0.089  0.002 | 1.67 [1.06-2.60]    3.82 [1.44-8.43] | 0.025    0.002 |
| Additional therapies, previous/concomitant  · Endocrine Therapy  · Other Targeted Therapy  · Chemotherapy | 0.89 [0.60-1.31]  0.92 [0.15-2.95]  0.57 [0.03-2.59] | 0.56  0.91  0.58 |  |  |
| **Chronic kidney disease (n=91, 1.9%);**  *Renal failure, Chronic kidney disease, Azotemia* | | | | |
| Age subgroup (Ref: <65 years)  · 65-74 years  · 75-84 years  · +85 years | 2.51 [1.52-4.14]  3.44 [1.98-5.91]  4.91 [1.65-11.79] | 0.0003  <0.0001  0.001 | 2.50 [1.52-4.13]  3.46 [1.98-5.94]  5.25 [1.77-12.66] | 0.0003  <0.0001  0.0008 |
| Additional therapies, previous/concomitant  · Endocrine Therapy  · Other Targeted Therapy  · Chemotherapy | 1.73 [1.14-2.67]  0.00 [0.00-383.48]  0.00 [0.00-1.50] | 0.011  0.98  0.97 | 1.77 [1.16-2.72] | 0.009 |

**Supplementary Figure S1. Disproportionality Analysis of Standardised MedDRA Queries (SMQs) for CDK4/6 Inhibitors (Palbociclib, Ribociclib, Abemaciclib) Using Reporting Odds Ratios (RORs)**


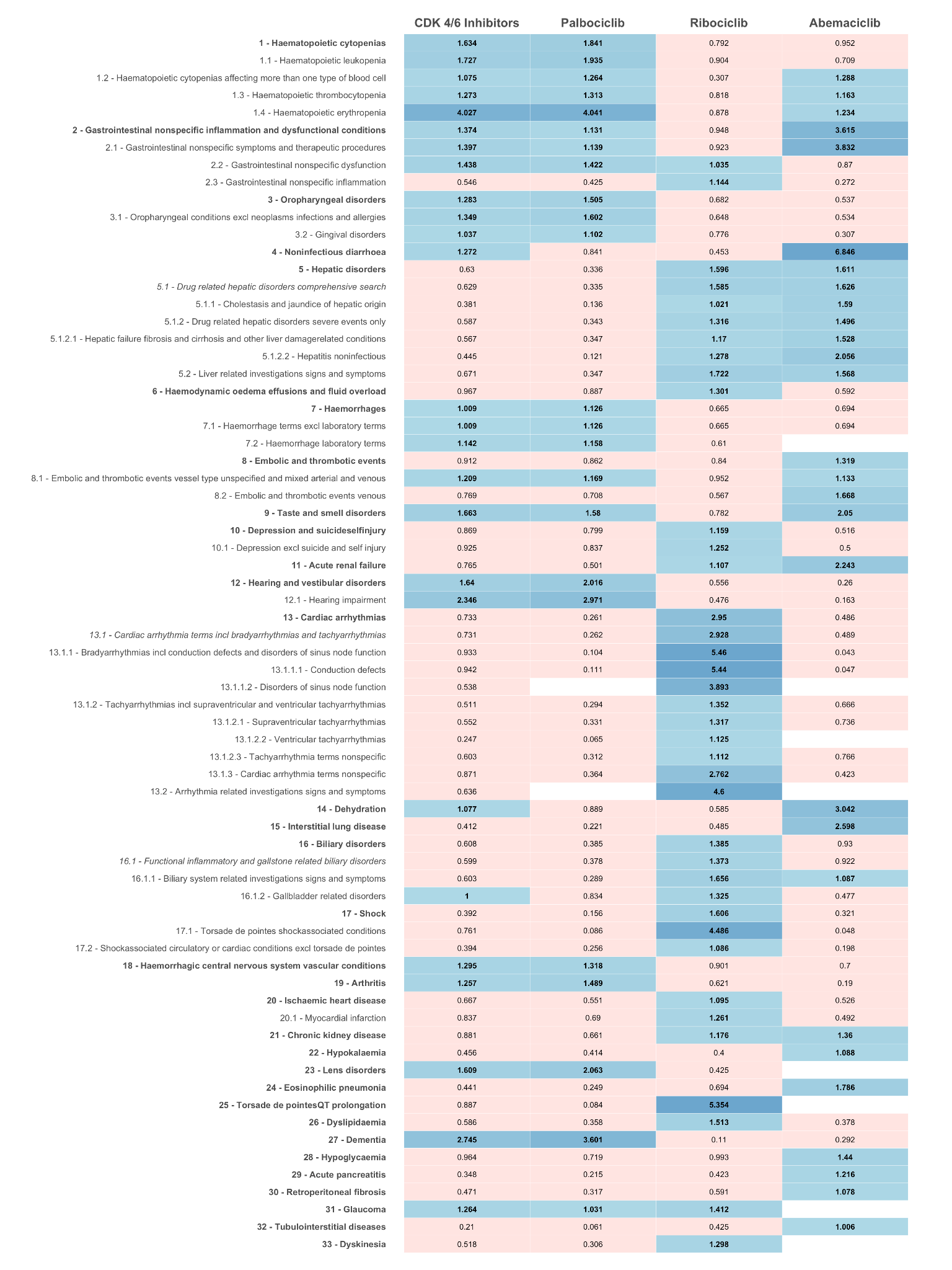

Supplement: Multimedia component 1 [file mmc1.docx]
